# Effects of Wet and Dry Finishing and Polishing on Surface Roughness and Microhardness of Composite Resins

**Published:** 2017-03

**Authors:** Negin Nasoohi, Maryam Hoorizad, Seyedeh Farnaz Tabatabaei

**Affiliations:** 1 Associate Professor, Department of Restorative and Cosmetic Dentistry, Islamic Azad University, Tehran, Iran; 2 Postgraduate Student of Restorative and Cosmetic Dentistry, Department of Restorative and Cosmetic Dentistry, Islamic Azad University, Tehran, Iran

**Keywords:** Composite Resins, Dental Polishing, Hardness

## Abstract

**Objectives:**

This study aimed to assess the effect of wet and dry finishing and polishing on microhardness and roughness of microhybrid and nanohybrid composites.

**Materials and Methods:**

Thirty samples were fabricated of each of the Polofil Supra and Aelite Aesthetic All-Purpose Body microhybrid and Grandio and Aelite Aesthetic Enamel nanohybrid composite resins. Each group (n=30) was divided into three subgroups of D, W and C (n=10). Finishing and polishing were performed dry in group D and under water coolant in group W. Group C served as the control group and did not receive finishing and polishing. Surface roughness of samples was measured by a profilometer and their hardness was measured by a Vickers hardness tester. Data were analyzed using two-way ANOVA (P<0.05).

**Results:**

The smoothest surfaces with the lowest microhardness were obtained under Mylar strip without finishing/polishing for all composites (P<0.0001). The highest surface roughness was recorded for dry finishing/polishing for all composites (P<0.0001). Dry finishing/polishing increased the microhardness of all composites (P<0.0001).

**Conclusions:**

Dry finishing and polishing increases the microhardness and surface roughness of microhybrid and nanohybrid composite resins.

## INTRODUCTION

Composite resins are increasingly used for direct restoration of teeth due to their optimal esthetics, favorable physical and mechanical properties, availability of efficient bonding systems and public concerns regarding amalgam [1,2]. Composite resins are classified based on the type, distribution and size of their filler particles. Microhybrid and nanohybrid composites are extensively used in anterior and posterior teeth [3]. Smoothness of the surface of composite restorations affects their durability and esthetic appearance [4]. Rough surfaces are unesthetic and result in discoloration of restoration [5], plaque accumulation, development of secondary caries and gingival inflammation [6] and wear of the opposing teeth [7]. The composite surface in contact with the Mylar strip is adequately smooth; however, due to high resin content, this surface is susceptible to wear. Moreover, intraoral finishing and polishing should be necessarily performed [8].

Hardness of composite resins is another important property related to the degree of polymerization of material, which affects the resistance of composite to wear as well as the wear of the opposing teeth or restorations [9]. The process of finishing can cause scratches in low-hardness composites. These scratches decrease the fatigue resistance of material and lead to restoration fracture and eventual failure [10]. Method of finishing and polishing significantly affects the esthetic appearance and durability of composite restorations [1].

Finishing is performed to create an anatomical shape and remove excess restorative material. Polishing is performed to increase the shine of restoration and yield a natural look resembling enamel [11]. Several studies have evaluated the effect of different finishing and polishing procedures on surface roughness and hardness of composites [12,13]. However, there is no consensus on the effect of conduction of finishing and polishing under water coolant on surface characteristics of composites. Thus, this study aimed to assess the effect of wet and dry finishing and polishing on surface hardness and roughness of commercially available microhybrid and nanohybrid composites.

## MATERIALS AND METHODS

### Preparation of samples:

Table 1 shows the composite resins used in this study. Finishing and polishing for experimental groups were conducted using Sof-Lex Pop-On Discs (3M ESPE, St. Paul, MN, USA) and aluminum oxide discs including coarse (60μm), medium (40μm), fine (24μm) and ultrafine (8μm) grit sizes. Thirty samples were fabricated of each composite resin using a metal mold measuring 10mm in diameter and 1mm in thickness.

**Table 1:** Composite resins used in the study according to the information provided by the manufacturers

**Material (Manufacturer)**	**Type**	**Matrix**	**Average particle size**	**Filler type**	**Filler loading vol%**	**Filler loading wt%**	**Shade**	**Batch number**
Aelite Aesthetic Enamel (BISCO, Schaumburg, USA)	Nanohybrid composite	Ethoxylated Bis-GMA, TEGDMA	0.5–2μm 0.05μm	Glass filler, amorphous silica	53	73	A2	1600004511
Aelite All Purpose Body (BISCO, Schaumburg, USA)	Microhybrid composite	Ethoxylated Bis-GMA, TEGDMA	0.04–0.7μm	Glass filler, amorphous silica	55	76	A2	1600004474
Grandio (Voco, Cuxhaven, Germany)	Nanohybrid composite	Bis-GMA, dimethacrylate, UDMA, TEGDMA	1μm, 20–50nm	Ba-Al-borosilicate glass filler, nanofiller (SiO2)	71.4	87	A2	1536078
Polofil supra (Voco, Cuxhaven, Germany)	Microhybrid composite	Bis-GMA, TEGDMA, UDMA	0.04–5μm	Glass filler, silica	60	76.5	A2	1408140

Composites were applied to molds and placed between two transparent Mylar strips. A glass slab was also placed on top of the upper Mylar strip and a constant pressure was applied in order for the excess composite to leak out. Next, the samples were light-cured for 20 seconds according to the manufacturer’s instructions using a quartz tungsten halogen light curing unit (Demetron LC; Kerr Corporation, Middleton, WI, USA). The intensity of light was measured by a radiometer (Model 10; Kerr Demetron, Danbury, CT, USA) prior to each time of use to ensure it was not less than 600 mW/cm^2^. Immediately after curing, the samples were removed from the mold and were randomly divided into three groups as follows:

Group C: This group received no finishing or polishing after removing the Mylar strip and served as the control group.

Group W (wet finishing and polishing): In this group, the samples were subjected to finishing and polishing using coarse, medium, fine and super fine aluminum oxide discs, respectively under water coolant provided by a water syringe held by a second operator with a flow rate of 20 cc/minute.

Group D (dry finishing and polishing): The samples in this group were subjected to finishing and polishing using coarse, medium, fine and super fine aluminum oxide discs, respectively without water coolant. After using each disc, the samples were rinsed for 10 seconds to remove debris and dried for 5 seconds.

Discs were discarded after one time of use and each disc was used for 20 seconds with mild pressure and planar movement in a low-speed (5000rpm) hand piece (Ti-Max Electric hand piece; NSK, Tokyo, Japan). All phases of finishing and polishing were performed by the same operator, who was blinded to the group allocation of samples. After finishing and polishing, all samples were rinsed and dried. The samples were then incubated at 37°C for seven days prior to measurement of surface roughness and hardness [14,15].

### Measurement of surface roughness:

The mean surface roughness was measured by a profilometer (TR 200 Surface Roughness Tester; TIME Group, Pittsburgh, PA, USA) with a tracing length of 2mm and 0.25mm cut-off. Tracing was performed in triplicate for each sample and the mean value was calculated [16].

### Measurement of microhardness:

Microhardness was measured using a Vickers hardness tester (D-89610; Bareiss Prüfgerätebau GmbH, Oberdischingen, Germany). Three indentations were made in each sample by applying 200g load within 15 seconds, and the mean value was calculated. A minimum of 1mm distance was considered between indentations [16].

### Statistical analysis:

Surface roughness and microhardness data were analyzed using two-way ANOVA followed by one-way ANOVA and Tukey’s multiple comparisons test. Level of significance was set at P<0.05.

## RESULTS

Table 2 shows the surface roughness values of composite resins subjected to different finishing and polishing systems. According to two-way ANOVA, the interaction effect of type of composite and treatment on roughness values was significant (P<0.001). Among all composites, the surface of group C samples, which received no finishing and polishing, showed significantly lower surface roughness than groups W and D (P<0.001).

**Table 2:** Mean surface roughness (Ra, μm) and Vickers microhardness (kg/mm^2^) values and standard deviations for the tested materials and polishing procedures

**Composite resins**	**Surface roughness values**			**Vickers microhardness values**		

	**Group C**	**Group W**	**Group D**	**Group C**	**Group W**	**Group D**
Aelite Aesthetic Enamel	0.02±0.01^Aa^	0.11± 0.01^Ab^	0.15± 0.01^Ac^	61.00± 2.06^Aa^	75.68± 2.09^Ab^	94.37± 2.99^Ac^
Aelite All Purpose Body	0.04±0.01^Aa^	0.13± 0.02^Ab^	0.16± 0.01^Ac^	63.97± 2.54^Aa^	78.20± 2.23^Ab^	96.78± 2.10^Ac^
Grandio	0.05±0.01^Aa^	0.32± 0.02^Bb^	0.43± 0.02^Bc^	115.09±6.56^Ba^	163.75± 2.86^Bb^	199.92± 4.47^Bc^
Polofil Supra	0.03±0.01^Aa^	0.13±0.01^Ab^	0.17±0.01^Ac^	61.23± 1.92^Aa^	76.15± 2.33^Ab^	96.20± 3.75^Ac^

C: Control, W: Wet finishing/polishing, D: Dry finishing/polishing

Means followed by different lowercase letters show statistically significant differences between them, as compared in rows.

Means followed by the same uppercase letters do not show statistically significant differences between them, as compared in columns.

For all composite samples, the surface roughness values for group W were significantly higher than those for group C (P<0.001), and the values in group D were significantly higher than those in group W (P<0.001).

In groups W and D, Grandio samples showed significantly higher roughness values compared to other composite resins (P<0.001). This difference was not significant in group C (P=0.111). The difference in surface roughness values for Aesthetic Enamel, All Purpose Body and Polofil Supra was not significant in group C (P=0.111), group W (P= 0.063) or group D (P=0.794).

Table 2 shows the surface hardness values of composite resins subjected to different finishing and polishing systems. According to two-way ANOVA, the interaction effect of composite type and treatment on hardness values was significant (P<0.001). Among all composites, the surface of group C samples, which received no finishing and polishing, showed significantly lower hardness values than groups W and D (P<0.001). For all composite samples, the surface hardness values for group W were significantly higher than those for group C (P<0.001), and the values for group D were significantly higher than those for group W (P<0.001).

Grandio samples showed significantly higher hardness values in all groups compared to other composite resins (P<0.001). The difference in surface hardness values for Aesthetic Enamel, All Purpose Body and Polofil Supra was not significant in group C (P=0.317), group W (P= 0.231) or group D (P=0.413).

## DISCUSSION

This study assessed the effect of dry and wet finishing and polishing on surface roughness and hardness of four microhybrid and nanohybrid composites. The results showed that finishing and polishing without water coolant increased the surface roughness and hardness of composite samples.

Finishing and polishing methods undergo constant modifications to improve durability and esthetic appearance of tooth-colored restorations [1]. Surface roughness of composite resins depends on several intrinsic and extrinsic factors. Intrinsic factors include type of material, type of filler, shape, size and distribution of filler particles, degree of polymerization, resin matrix composition and durability of filler/matrix bond [17]. Extrinsic factors are related to the method of finishing and polishing and include the flexibility of polishing tool, hardness of abrasive particles, geometrical shape of polishing tool and its method of application [18].

In the current study, the lowest surface roughness in all composite samples was found in the surface in contact with the Mylar strip (group C), which was in agreement with the findings of a previous study [14]. This finding can be explained by the fact that finishing and polishing remove matrix between filler particles and resultantly, filler particles sticking out of the composite surface increase the surface roughness [19]. In our study, Grandio nanohybrid composite had the highest surface roughness after both wet and dry finishing and polishing because this composite contains 1μ glass particles that stick out from the surface and increase surface roughness [20]. Jung et al. [13] evaluated several nanohybrid composite resins and found that only Grandio composite had higher surface roughness than hybrid composites.

In our study, the surface roughness of all composite samples was higher following dry finishing and polishing compared to those subjected to wet finishing and polishing. In dry finishing and polishing, composite surface roughness may increase because the abrasive particles separated from the polishing tool may be embedded into the composite surface. Moreover, accumulation of separated particles on the surface of polishing tool can decrease its efficiency in smoothing the surface [21]. On the other hand, heat generated during dry finishing and polishing is high and can degrade the filler/matrix bond and result in separation of filler particles from the matrix and subsequently increase the surface roughness [22].

Bacterial accumulation significantly increases when the composite surface roughness exceeds 0.2μ [23]. However, according to a study by Bollen et al, [24] patients cannot recognize surface roughness less than 0.3μ. In our study, dry finishing and polishing increased the surface roughness of all composites; however, this increase did not reach the critical level for bacterial accumulation or the clinically perceivable level by patients in any composite except for Grandio. This finding indicates that no significant difference exists between composites clinically in terms of surface roughness. Chung [25] showed that restorations with surface roughness less than 1μ appear perfectly smooth. In our study, all composite samples showed surface roughness less than 1μ.

Composite hardness depends on several factors such as type and shape of filler particles, their composition and distribution, percentage of filler particles, and type of resin [26]. Reduction in hardness of filler particles directly decreases the hardness of composite [27]. In our study, the lowest hardness in all composite resin samples belonged to the group cured in contact with Mylar strip (group C), which was in line with the findings of previous studies [14,16] because this layer contains high resin content and has poor mechanical properties [28]. In our study, Grandio nanohybrid composite yielded the highest hardness among tested composites after both dry and wet finishing and polishing because this composite has 87wt% filler content, which is higher than that of other composites evaluated in our study. Increase in filler content enhances the hardness of composites [27]. Similarly, Cekic-Nagas et al. [29] reported that Grandio composite samples had the highest microhardness among five resin composites. In our study, hardness of all composite samples increased by dry finishing and polishing. The diametral tensile strength and hardness of composite increase by raising the temperature up to 60°C, which is due to increased cross-linking between polymer chains [30]. Infrared tomography assessments have shown that the temperature at the surface of composite subjected to dry finishing and polishing is 140°C or higher [31]; such a temperature rise increases cross-linking and hardness because this temperature is higher than the glass-transition temperature of resin content [32]. This temperature rise is not hazardous for dental pulp because composites are heat insulator, and the generated heat during dry finishing and polishing is confined to the composite surface such that at 0.2mm depth from the composite surface, temperature does not exceed 10°C [33].

Contrary to the results of the current study, Dodge et al. [21] showed that dry finishing and polishing decrease the surface roughness of microfilled composites while increase their surface hardness, which could be related to difference in filler content and organic matrix composition.

Marigo et al. [34] showed that characteristics of finishing and polishing tools such as their flexibility, shape and hardness of abrasive particles affect the resultant surface roughness of composite. Since it has been reported that flexible aluminum oxide discs are ideal for obtaining a smooth composite surface [35], we used Sof-Lex aluminum oxide discs in this study. Fruits et al. [36] reported that planar movement yields the lowest surface roughness following finishing and polishing of composite. Thus, we finished and polished composite samples using planar movement.

In our study, finishing and polishing were performed manually in order to better simulate the clinical setting. Jones et al. [37] indicated that applied load and speed of finishing and polishing are widely variable among different operators. In our study, one operator performed finishing and polishing of all composite samples. According to Heintze et al, [38] finishing and polishing for 60 seconds decrease the surface roughness to a level below the critical threshold for bacterial accumulation. In our study, each sample was finished and polished for 80 seconds and this time was controlled by a chronometer.

## CONCLUSION

Considering the limitations of this in vitro study, it can be concluded that dry finishing and polishing could increase the surface roughness and microhardness of microhybrid and nanohybrid composite resins.
